# 

**Published:** 1835

**Authors:** 


### RECEIPTS FOR NINTH VOLUME.

The following Physicians have paid for the 9th volume of the Journal in advance. We design this acknowledgment to stand as receipts—hence no written ones will be sent unless especially requested. Hereafter we expect to append an account of the money received within a quarter to the end of the following number. We hope we shall be able to place a majority of the credits in the early numbers of the next volume.

JOHNSON & WOOD.

A

Aikin, J. E. Greensburgh, Ky. $1,50
Albertson, Benj. Salem, Ia.
Allen, P. Kinsman, O.
Alexander, Harman Palestine, Ill.

B

Beach, M. B. Waterford, Pa.
Beck, J. M. Bethel, O.
Bennett, —— Williamsville, O.
Bissell, H. Wooster, O.
Blackmore, J. A. Shelbyville, Tenn.
Blackstone, W. Waverly, O.
Beach, H. Fowler, O.
Bonnell, W. W. Greenford, Ia.
Bradley, M. B. Waterford, Pa.
Bragdon, G. H. Mear's Farm, O.
Brashaw, J. Nine-mile-prairie, Ill.
Brashear, A. Vermillionville, La.
Brown, H. Newtown, O.
Burns, G. H. Bricksville, Ala. $1,00
Burrett, G. Gillead, O.

C

Campbell, F. W., H. Court-house, A.T.
Camby, J. T. Crawfordsville, Ia.
Cardwith, J. W. Paris, Ill.
Carter, J. Versailles, Ky.
Carpenter, E. G. Chester, O.
Cartwright, S. A. Natchez, Miss.
Carver, H. Economy, Ia.
Chandler, W. B. Warsaw, Ky.
Charters, W. Lebanon, O.
Christian, —— Clarksburgh, Va.
Clapp, A. New Albany, Ia.
Clark, A. Gambier, O. $2,00
Cochran, J. S. Sandusky City, O.
Colby, J. Cincinnati, O.
Cole, J. Akron, O.
Cooms, A. S. Oakland, Ill.
Condwith, J. M. Paris, Ill.
Cooper, S. Stillville, O.
Crossfield, E. D. Red Lion, O.
Cunningham, F. A., W. Alexandria, O.

D

Dancy, C. F. M. Decatur, Ala.
Daniels, E. Reading, Pa.
Deadman, D. G. Lawrenceburgh, Ky.
Dellenbaugh, J., N. Georgetown, O.
Dill, A. S. Mayfield, O.
Dunlap, M. Greenfield, O.

E

Eastman, B. Paris, Ia.
English, J. West Liberty, Va.
Ewing & White, Franklin, Tenn.
Evans, D. T. Cincinnati, O.

F

Frazer, J. C. Cynthiana, Ky.
Francis, C. New York city.
Frierson, J. W. S. Columbia, Tenn.

G

Gano, J. E. Centreville, Ky.
Gordman, J. Clarksville, O.
Goodwin, H. Springfield, O.
Grooms, H. B. Elkton, Ky.

H

Harris, A. S. Greensborough, Ala.
Harrison, J. P. Cincinnati, O.
Hays, J. B. Columbia, Tenn.
Helmick, J. Zanesville, O.
Henderson, J. P. Newville, O.
Hendricks, —— South Bend, Ia.
Henderson, R. G. Lawrenceburg, Tenn.
Hildreth, S. P. Marietta, O.
Hodgen, R. Campbellsville, Ky.
Hood, A. Winchester, Ky.
Hughes, J. R. Oxford, O.
Hunt, F. W. South Bend, Ia.
Huntington, R. G. Ellsworth, O.
Hissy, C. G. Monroeville, Ia.

I

Ingalls, D. Jeffersontown, Ky.

J

Jackson, S. Northumberland, Pa.
Jacobs, F. Delhi, N. Y. $11,00
Jewett, H. Dayton, O.
Jewett, J. B. Madison, O.
Johnson, J. T., N. Richmond, O.

K

Kittredge, E. E., P. Assump., La.

L

Lancaster, J. Loretto, Ky.

Larue, J. J. Hodgensville, Ky.

Letcher & Walker, Richmond, Ky.

Lindsay, Wm. Richmond, Ia.

Lithicum, R. Lewisburgh, Ky.

Littleton, John Columbia, Tenn.

Loring, H. S. Russellsville, Ky.

M

Maddox, T. N. Louisville, Ky.
Martin, E. Bloomington, O.
Martin, L. New Liberty, Ky.
Mayfield, D. S Franklin, Tenn.
Mason, J. M. Cincinnati, O.
Mason, P. Connersville, Ia.
M'Crarey, J. M'Crarey's, Tenn.
M'Farland, J. B. Hamilton, O.
M'Guire, W. Williams' P.O., O.
M'Mechan, J. Trenton, O.
Micheaux, J. W. Moulton, Ala. $4,00
Mills, A. W. Winchester, Ky.
Morton, G. Decatur, O.

N

Norton, G. Decatur, O.
——, Drs. Norwich, O.

O

O'Brian, L. Franklin, Tenn.
O'Bryant, W. Clarksburgh, Ia.
Orr, J. Alexandria, Ky.
O'Ferrell, J. Piqua, O.

P

Palmer, S. Clinton, Ia.
Paramore, J. Eaton, O.
Parker, J. F. Shelbyville, Ky.
Pawling, Wm. Lexington, Ky.
Petit, —— Pittsburgh, Pa.
Preston, C. H., N. Franklin, O.
Price, A. B. Centreville, O.
Posey & Coleman, Livingston, Ala.

R

Ray, L. G. Paris, Ky.
Rhodes, D. W. Zanesville, O.
Riddell, J. L. Cincinnati, O.
Robertson, H. A. Yellow Creek, O.
Rogers, J. G. New Richmond, O.

S

Safford & Brown, Putman, O.
Saturwhyte & Whitney, Lexington, Ky.
Sexton, S. Greenville, O.
See, J. Salem, Ia.
Shreeve, J. Mt. Union, O. $2,00
Simmons, J. Pleasant Exchange, Tenn.
Smith, J. Paris, Ky.
Smith, E. Eagle, O.
Spafford, A. Cooperstown, N. Y.
Spindle, J. P. Mt. Pleasant, Tenn.
Sparks, J. H. Wilmington, O.
Stewart, J. H. Sydney, O.
Stevens, T. C. Geneva, O.
Steikel & Smith, Hennepin, Ill.
Stone, J. C. Cincinnati, O.
Sugg, H. H. Keysburgh, Ky.
Swan, J. S. Henderson, Ky.

T

Taylor, J. H. Harrodsburgh, Ky. $1,50
Taylor, T. P. Gosport, Ia.
Templeton, J. Centreville, O.
Thiel, C. Gratis, O.
Thompson, W. Bethel, O.
Thornton, S. S. Batavia, O.
Towler, T. S. Springfield, O.
Towns, J. M. Oakachickama, Miss.
Turnbull, C. Princeton, Miss.

V

Vandeveer, P. Middletown, O.
Vangorden, C. Warren, O.
Vaught, M. Shawneetown, Ill.

W

Walker, Jas. New Richmond, O.
Welsh, T. Wilmington, O.
Welsh, J. S. Waynesburgh, O.
White, J. B. Columbia, Ky.
Whitesel, J. M. Knightstown, Ia.
Williams, G. A. Adamsville, Ky.
Wilson, W. B. West Union, O.
Wilson, S. S. Clifton, O.
Winston, C. R. Columbia, Ky.
Wood, S. Morefield, O.
Woods & Faulk, Lebanon, Ky.

The proprietors would observe that, owing to the transfer of the Journal, this list may not embrace all the advance payments. If, however, any error should be detected by our subscribers, it will readily be corrected. One of the proprietors lost a letter, containing a remittance, a few days since, when he was leaving the Post-Office. He may yet find it, but should he not do so the money will be placed to the c[illegible]t of the proper person when further information is received. He remembers the residence of the individual, but has forgotten his name.

THE WESTERN JOURNAL OF THE MEDICAL AND PHYSICAL SCIENCES, is printed quarterly by N. S. Johnson, No. 133, Main St., Cincinnati, for the proprietors, Dr. C. R. COOPER and SILAS REED.

TERMS — THREE DOLLARS in advance, THREE DOLLARS AND FIFTY CENTS within six months, or FOUR DOLLARS at the end of the year.

